# The role of neutrophils in immune dysfunction during severe inflammation

**DOI:** 10.1186/s13054-016-1250-4

**Published:** 2016-03-23

**Authors:** Pieter H. C. Leliefeld, Catharina M. Wessels, Luke P. H. Leenen, Leo Koenderman, Janesh Pillay

**Affiliations:** Department of Surgery, University Medical Center Utrecht, Utrecht, The Netherlands; Department of Respiratory Medicine, University Medical Center Utrecht, Utrecht, The Netherlands; Laboratory of Translational Immunology, University Medical Center Utrecht, Utrecht, The Netherlands; Department of Anesthesiology and Critical Care, University Medical Center Utrecht, Utrecht, The Netherlands

## Abstract

Critically ill post-surgical, post-trauma and/or septic patients are characterised by severe inflammation. This immune response consists of both a pro- and an anti-inflammatory component. The pro-inflammatory component contributes to (multiple) organ failure whereas occurrence of immune paralysis predisposes to infections. Strikingly, infectious complications arise in these patients despite the presence of a clear neutrophilia. We propose that dysfunction of neutrophils potentially increases the susceptibility to infections or can result in the inability to clear existing infections. Under homeostatic conditions these effector cells of the innate immune system circulate in a quiescent state and serve as the first line of defence against invading pathogens. In severe inflammation, however, neutrophils are rapidly activated, which affects their functional capacities, such as chemotaxis, phagocytosis, intra-cellular killing, NETosis, and their capacity to modulate adaptive immunity. This review provides an overview of the current understanding of neutrophil dysfunction in severe inflammation. We will discuss the possible mechanisms of downregulation of anti-microbial function, suppression of adaptive immunity by neutrophils and the contribution of neutrophil subsets to immune paralysis.

## Background

Severe inflammation induced by trauma, sepsis or ischemia/reperfusion injury is known to contribute to devastating complications such as acute respiratory distress syndrome (ARDS) and (multiple) organ failure [1]. This has been attributed to microvascular dysfunction, tissue damage and dysregulation of metabolism caused by severe inflammation [2]. In recent years, however, it has been recognised that severe systemic inflammation can also result in a profound ‘compensatory’ down-regulation of immune responses, rendering the host susceptible to infections or unable to clear existing infections [3]. Although conceivably an evolutionarily preserved response to protect the host from immune-mediated tissue damage, downregulation of anti-microbial immunity creates an unwanted consequence: susceptibility to bacterial infections such as caused by *Staphylococcus aureus*, *Pseudomonas aeruginosa* and *Escherichia coli* as well as opportunistic fungal infections such as (disseminated) candidiasis [4–6]. In addition, reactivation of viruses such as cytomegalovirus are found in critically ill patients [7]. These findings clearly indicate that both the innate and the adaptive immune system are dysfunctional in these patients. Nosocomial infections in critically ill patients are associated with an increased length of hospital stay, increased health care costs and profound additional morbidity and mortality [8].

Neutrophils, effector cells of the innate immune system, are abundantly present in the circulation and comprise up to 50–70 % of total circulating leukocytes in humans. The enhanced frequency and severity of bacterial and fungal infections in patients with congenital neutrophil disorders demonstrate that neutrophils are indispensable for adequate protection against microbes [9]. Patients suffering from leucocyte adhesion deficiency (LAD)-I are at risk for development of necrotizing infections and sepsis because of inadequate neutrophil transendothelial migration to the site of infection [10]. The Chediak-Higashi syndrome and chronic granulomatous disease (CGD) underscore the eminent importance of intracellular bacterial killing by neutrophils. Chediak-Higashi syndrome is caused by a mutation in the *LYST* gene, which encodes a lysosomal trafficking regulator [11]. The mutation leads to the absence of a proper formation of phagolysosomes. Patients suffering from Chediak-Higashi are extremely susceptible to pyogenic infections and this syndrome is usually fatal before the age of 10 [11]. CGD is characterised by a defect in production of the bactericidal reactive oxygen species (ROS) due to defective NADPH oxidase and results in recurrent infections, reducing life-expectancy significantly [12]. In murine models of sepsis, knockout of essential neutrophil antimicrobial functions leads to rapid death. For instance, mice lacking the neutrophil granule proteins myeloperoxidase or elastase die more rapidly from sepsis [13, 14]. Apart from the severe phenotypes seen in patients with inborn errors and murine knockout models, more subtle effects were seen in a murine sepsis model where rapid death coincided with inadequate phagosomal acidification of neutrophils after phagocytosis [15]. These studies highlight the generally accepted importance of neutrophils in antimicrobial defence in acute inflammatory models. In addition, they demonstrate disturbances in the anti-microbial functionality of these cells during severe inflammation.

In this review we will discuss neutrophil functions required for adequate microbial defence and the mechanisms leading to neutrophil-mediated immune dysfunction.

## Functions of neutrophils associated with anti-microbial defence

### Chemotaxis

The controlled process of phagocytosis and killing of microbes by neutrophils firstly requires chemotaxis towards the site of infection. Chemotaxis is the propensity of cells to migrate in the direction of gradients of chemotactic stimuli [16]. The ability to adequately sense chemotactic gradients is one of the final capabilities acquired by neutrophils during maturation in the bone marrow and this functionality appears to be the most sensitive to perturbations in vivo and in vitro [17]. Impairment of chemotaxis has been described in a wide variety of diseases associated with increased susceptibility to infections: diabetes mellitus, viral infections (influenza), cytomegalovirus, HIV and tropical diseases (malaria) [18–22]. In sepsis, chemotaxis of neutrophils is impaired through various mechanisms [23–25]. Interleukin (IL)-33 limits this impairment by preventing downregulation of CXCR2 and improves outcome in a murine model [26]. In humans, extensive research has focused on the chemotactic capacity of neutrophils from burn patients. It has been shown that neutrophils from thermally injured subjects are characterised by impaired chemotaxis, both in vivo in the tissue and in vitro, towards the bacterial peptide fMLF, which is believed to contribute to the increased susceptibility to infections in this group of patients [27, 28].

### Intracellular killing

Once neutrophils have found and recognised a pathogen, phagocytosis can take place and subsequent bacterial killing occurs in the phagolysosome. Neutrophils possess two separate but intercalating anti-microbial mechanisms, one dependent on oxygen and the other independent of it. Although categorisation of killing mechanisms in this manner creates a comprehensive understanding, it does not reflect the in vivo situation in which both systems operate simultaneously. Furthermore, it is likely that the individual significance of both killing mechanisms shifts during the course of inflammation. This is due to fluxes in oxygen demand and supply caused by dynamic tissue perfusion and oxygenation during the inflammatory response [29].

The oxygen-dependent mechanisms are mediated by ROS downstream of O_2_^−^ formed by the NADPH oxidase complex [30]. In short, upon activation of a neutrophil, either via ingestion of bacteria or by extracellular stimuli, the NADPH oxidase complex is assembled from both cytosolic and membrane-bound components [31]. The active oxidase complex transports electrons from cytosolic NADPH across the membrane to the electron acceptor, molecular oxygen, generating superoxide anion [29]. This is in turn metabolises in the phagosome into highly bactericidal end products, such as hydroxyl radical, hydrogen peroxide and hypochlorous acid [31]. In sterile inflammation, such as trauma or acute liver failure, neutrophils are known to produce elevated levels of spontaneous ROS [32, 33]. Furthermore, ROS production in these patients in response to a stimulus is strongly elevated in comparison with that found in neutrophils from healthy controls; a process generally referred to as priming [27, 34–36]. Uncontrolled release of ROS by neutrophils accumulating in vascular beds can contribute to loss of endothelial barrier integrity and subsequent vascular leakage, predisposing patients to organ injury as a result of pro-inflammatory complications (acute lung injury, ARDS) [37, 38]. This hypothesis is in line with the findings of increased ROS production in trauma patients developing ARDS in comparison with control trauma patients [39]. In addition, the observation that neutrophils from patients with fatal sepsis are characterised by markedly increased production of ROS compared with survivors is noteworthy [40].

Granule products comprise the backbone of non-oxidative killing by neutrophils [41]. The azurophilic granule is a reservoir of serine proteases: neutrophil elastase, cathepsin G, proteinase 3, and azurocidin [42]. These digestive proteases are delivered into the phagolysosome upon fusion of granules with a phagosome containing bacteria. During maturation of the phagolysosome the intraphagosomal pH is rigorously altered. The early shift of intraphagosomal pH towards an alkaline level (pH 8.5–9.5) due to dismutation of O_2_^−^ provides the initial milieu for the proper activation of proteases, leading to optimal microbicidal and digestive function of these enzymes [43]. Concomitant with the waning of production of ROS the phagosome progressively acidifies, coinciding with granule–phagosome fusion. These granules contain the Na^+^/H^+^-antiporter V-ATP-ase, which is responsible for pumping of protons into the phagosome [44–46]. Neutrophils of burn-injured patients are characterised by dysfunctional pH control of their phagolysosomes since these patients fail to demonstrate transient phagosomal alkalinisation in the first 5 minutes and acidify promptly after ingestion of bacteria [47]. This situation might lead to improper activation of the proteases and impaired killing of ingested microbes. On the other hand, deficient acidification of peritoneal neutrophils in a murine model of sepsis was associated with increased mortality [15]. These findings demonstrate the importance of adequate intraphagosomal pH regulation for microbial control.

The presence and proper function of granules intracellulary are crucial as these organelles supply neutrophils with an arsenal of antimicrobial mechanisms. However, uncontrolled activation of neutrophils in an inflammatory microenvironment can lead to collateral tissue damage by excessive extracellular degranulation and the release of neutrophil proteases. Neutrophil extravasation, homing and activation are mediated by activation of several surface receptors, including β2 integrins, complement receptors, Fcγ-receptors, and formyl peptide receptors. Uncontrolled activation of neutrophils is mediated through these same receptors by responding to aberrant production of chemokines, cytokines and release of extracellular peptides [48]. During this process granules fuse with the plasma membrane, releasing their content into the environment [49]. More tissue damage will lead to increased influx and activation of neutrophils, which then leads to a vicious cycle of tissue destruction [50].

### Neutrophil extracellular traps

In addition to conventional intracellular killing and degradation of individual bacteria, the concept of extracellular killing by neutrophils using neutrophil extracellular traps (NETs) has received much attention during the past decade [51, 52]. NETs consist of fibrils formed by active expulsion of DNA, chromatin and granule proteins from neutrophils [52, 53]. They are formed in response to a variety of pro-inflammatory stimuli of which IL-8, tumour necrosis factor-alpha and lipopolysaccharide are the most relevant [54]. During formation of NETs neutrophils die and this process is generally referred to as NETosis. This form of cell death is dependent on the NADPH-oxidase complex since neutrophils treated with the pharmacological NADPH-oxidase inhibitor DPI and CGD patients are unable to release NETs [53]. In vitro NETs were shown to be a cell-death-associated event occurring hours after stimulation [53]. However, intravital microscopy revealed viable neutrophils after formation of NETs and the resulting anuclear neutrophils were subsequently capable of phagocytosis and formation of mature phagosomes. These data indicate that NETosis does not necessarily result in cell death [55]. The direct bactericidal properties of NETs are a topic of discussion, and prevention of bacterial dissemination in vivo might be their main function [56]. Apart from this antimicrobial function, the cytotoxicity of NETs can be harmful to the host if their release is inappropriately controlled. NETs are released following sepsis, trauma and ischemia–reperfusion injury and a growing body of evidence shows they can contribute to tissue destruction, as reviewed by Liu et al. [57]. The potential of NETs to cause tissue destruction was elegantly demonstrated in a murine model of primary graft-dysfunction after lung transplantation [58]. In addition, several studies argue that NETs might be involved in triggering auto-immune diseases since auto-antibodies against NET constituents (e.g. DNA) are often found in these diseases [59, 60]. Although NETs have firmly established their tissue-damaging properties, scepticism still exists about the in vivo anti-microbial relevance of NETs [61].

## Neutrophil dysfunction in acute inflammation

The mechanisms involved in adequate anti-microbial defence can also disrupt subsequent immunity. This is caused by aberrant control of their own essential antimicrobial arsenal, such as: (1) auto- and paracrine cleavage of essential surface receptors; (2) downregulation of surface receptors and signalling pathways in non-resolving inflammation or during a second microbial hit following initial sterile inflammation (damage-associated molecular pattern (DAMP)–microbe-associated molecular pattern (MAMP) interference); and (3) the release of neutrophil populations with decreased microbicidal properties. In addition, neutrophils in inflammatory conditions can affect other immune cells and contribute to immune paralysis of the adaptive immune system.

### Proteolytic cleavage by neutrophil-derived proteases and downregulation of immune receptors

**Fig. 1:**
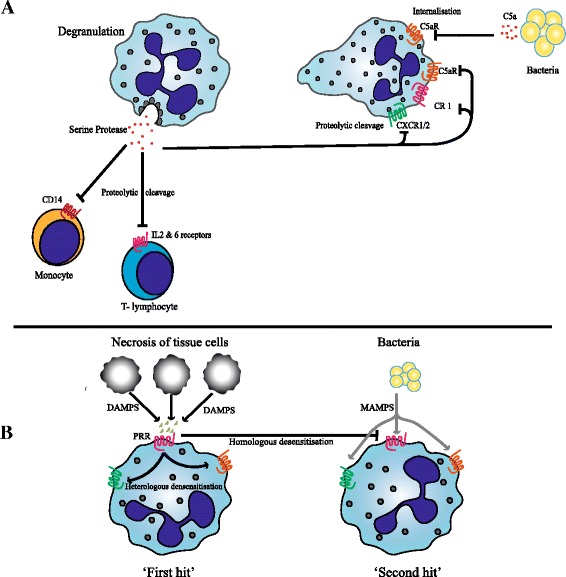
Downregulation of immune receptors by serine proteases from degranulated neutrophils and desensitisation by pattern recognition receptors. **a** Schematic representation of downregulation of receptors on neutrophils, monocytes and lymphocytes during inflammation due to cleavage by neutrophil serine proteases after degranulation. Binding of C5a to neutrophils results in internalisation of C5aR. Decreased expression of these receptors impairs neutrophil effector functions during subsequent challenges. **b** Biological mimicry between DAMPs and MAMPs. Danger signals derived from necrotic tissue cells ("*First hit*") bind to pattern recognition receptors (*PRRs*) and limit subsequent responses to microbial signals ("*Second hit*") through homo- and heterologous desensitisation. *DAMP* damage-associated molecular pattern, *IL* interleukin, *MAMP* microbe-associated molecular pattern

Serine proteases released by neutrophils influence the expression of receptors critical to neutrophil–microbial interactions (Fig. 1a). Apart from stimulatory effects through serine protease activated receptors (PARs), they can downregulate immune responses by cleaving essential receptors on the surface of both adaptive and innate immune cells [62]. For instance, neutrophil elastase cleaves CXCR1, a receptor for IL-8, on the surface of neutrophils [63, 64]. This mechanism is relevant during acute inflammation in which circulating neutrophils from trauma and sepsis patients selectively downregulate CXCR2, the only other neutrophil receptor for IL-8 [65, 66]. Tarlowe et al. [67] provided evidence that downregulation of this receptor preceded the occurrence of pneumonia in critically ill trauma patients. Downregulation of CXCR2 and cleavage of CXCR1 would result in severe hyporesponsiveness to IL-8, an important neutrophil chemoattractant.

Furthermore, neutrophil serine proteases can cleave complement receptors such as the CR1 receptor (CD35) and C5aR (CD88) on neutrophils [68, 69]. These receptors are important as they mediate chemotaxis, degranulation and proper recognition of opsonised microbial targets by CR1 and C5aR, respectively [70]. During inflammation, decreased expression of C5aR is seen due to engagement and subsequent internalisation. This can result in a profound defect in neutrophil phagocytosis of subsequent pathogens as C5a-induced chemotaxis is important for neutrophils to find opsonised targets [71]. Proteases not only inhibit the function of neutrophils, they can also affect monocytes in the micro-environment. Neutrophil elastase cleaves CD14, a receptor necessary for the high affinity recognition of lipopolysaccharide by TLR4, thereby impairing proper bacterial recognition by monocytes [72]. Lastly, elastase and cathepsin G mediate shedding of cytokine receptors for IL-2 and IL-6 on T lymphocytes [73].

### DAMP–MAMP interference

Trauma and ischemia/reperfusion injury can evoke the release of large amounts of cellular components from necrotic cells. These intracellular constituents are known as damage-associated molecular patterns (DAMPS). They are host-derived and serve as important pro-inflammatory non-microbial stimuli after injury [74]. Since the development of the ‘danger hypothesis’ by Matzinger [74], a large number of studies have focussed on molecules driving this response. The most extensively studied DAMPS are high-mobility group box 1, heat shock proteins, ATP, uric acid, formylated peptides from mitchondria and mitochondrial DNA [75–80]. Inflammation induced by pathogens on the other hand is mediated through microbial constituents referred to as microbe-associated molecular patterns (MAMPS), which resemble DAMPS and, importantly, share similar pattern recognition receptors (PRRs) on the neutrophil [81]. This biological mimicry and utilisation of similar receptors creates a problem for the immune system since injury (DAMPS) causes downregulation of many of these receptors by hetero- and homologous desensitisation. This can render neutrophils unable to mount an adequate response to a subsequent microbe (MAMP) (Fig. 1b). To illustrate the relevance of this phenomenon, Zhang et al. [80] showed the release of vast amounts of mitochondrial formylpeptides into the circulation of major trauma patients. These molecules stimulate neutrophils via formyl peptide receptor 1 (FPR1), an important receptor in recognizing microbes that produce danger signals by release of formyl-peptides [80] (Fig. 1b). It was shown that heterologous desensitisation of chemokine receptors and homologous desensitisation of FPR1 occurred simultaneously, predisposing trauma patients to infection [82].

### Release of incompetent neutrophil populations

**Fig. 2:**
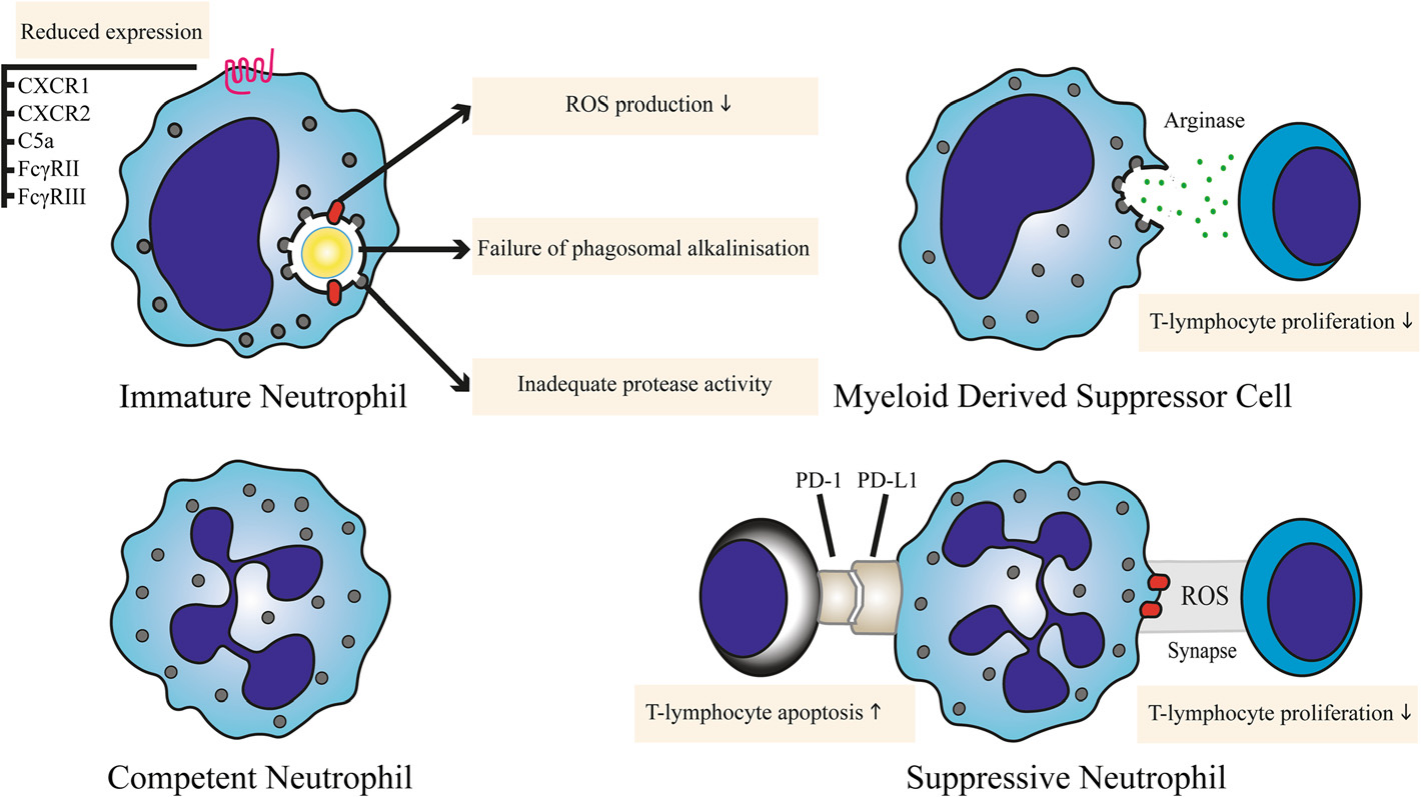
Circulating neutrophil subsets in severe inflammation. At least four types of neutrophils circulate in the bloodstream of patients during severe inflammation: immature, competent and suppressive neutrophils and myeloid-derived suppressor cells. Mechanisms contributing to immune dysfunction are displayed for neutrophils from different subsets. *ROS* reactive oxygen species

Much of the work detailed in the previous sections did not take into account the variations in functional phenotypes that appear in the circulating neutrophil compartment during severe inflammation. After maturation neutrophils are retained in the bone marrow via expression of chemokine receptor CXCR4 (ligand CXCL12), whilst CXCR2 (ligands IL-8/CXCL1 and 2) controls release into the peripheral blood. Inflammatory stimuli can induce the release of neutrophils by disrupting the balance in CXCR4/CXCL12 signalling through various mechanisms [60]. In severe inflammation large numbers of neutrophils are released into the circulation from the bone marrow post-mitotic pool as well as from the marginated pool (i.e. neutrophils not freely circulating but attached to the microvasculature) [83]. Under these conditions we have previously shown that peripheral neutrophils consist of heterogeneous subsets with different priming states and function [84]. During severe inflammation a large number of immature or banded cells appear in the circulation and even neutrophil progenitor cells can be identified. As a result, persistent severe inflammation might lead to "bone marrow exhaustion" of neutrophils, which is thought to inevitably result in a state of compromised innate immunity [85]. At present, however, it is unclear how to interpret the presence of immature cells in the bloodstream in response to inflammation. It might be a compensatory response initiated by the depletion of mature neutrophils in the bone marrow or a dedicated inflammatory reaction to a bacterial stimulus. Our data support the first hypothesis since these immature neutrophils also show a pronounced decrease of various receptors in comparison with their mature circulating counterparts [84]. In addition to the IL-8 receptors (CXCR1 and CXCR2) and the C5a receptor, the Fc receptors (CD16 and CD32), which are important in pathogen recognition, phagocytosis and killing, are also downregulated on immature cells (Fig. 2) [84]. Relatively few studies have assessed the functionality of immature and progenitor neutrophils subsets in severe human inflammation. In septic patients, immature neutrophils were shown to have decreased phagocytic capacity [86]. Importantly, reduced phagocytosis and increased numbers of circulating neutrophil progenitors are both associated with poor outcome in septic patients as well as in patients with severe inflammation [87, 88].

## Suppression of adaptive immunity by neutrophils

Neutrophils have long been recognised as professional killers. Eradication of bacteria and fungi was thought to be their main task. Evidence is, however, accumulating that apart from their direct anti-microbial function, neutrophils participate in subsequent modulation of (adaptive) immune responses in severe inflammation [89–91]. Under these inflammatory conditions, neutrophils produce chemokines and secrete granule contents which can subsequently attract and modulate the function(s) of T cells both directly and indirectly [92, 93]. For instance, neutrophil elastase reduces expression of co-stimulatory molecules by dendritic cells, limiting maturation and induction of a proper Th1 response [94]. In addition, T cells in the inflammatory microenvironment may be affected by neutrophil elastase by cleavage of their IL-2 and IL-6 receptors (Fig. 1a) [95]. Another mechanism of immune-modulation was observed in macrophages after phagocytosis of apoptotic neutrophils. Under these conditions immune responses of macrophages shift towards a more anti-inflammatory cytokine profile [96]. Furthermore, neutrophils themselves have been shown to produce anti-inflammatory cytokines such as IL-1ra and IL-10 [97]. However, the evidence regarding IL-10 production by neutrophils is controversial, as it has only been shown in mice with mycobacterial infections [98]. In humans neutrophils are unable to produce IL-10 [99]. Direct regulation of T-cell responses by neutrophils is slowly becoming an established concept. A large body of evidence demonstrates that a heterogeneous group of immature mononuclear cells and neutrophils termed myeloid-derived suppressor cells (MDSCs) can suppress T-cell responses in several murine tumour models. In addition, these cells have been shown to play a role in various models of infectious diseases, organ transplantation and autoimmune diseases [100]. Identification of human immature granulocytic MDSCs has proven to be challenging though. In particular, their differentiation from mature neutrophil phenotypes seen in the blood during acute inflammation remains to be established, as we have reviewed in detail elsewhere [101]. The mechanisms by which MDSCs can suppress T cells include the expression and secretion of arginase-1, which depletes arginine from the microenvironment (Fig. 2) [102]. Depletion of L-arginine, which is an essential amino acid, results in cell cycle arrest of T cells in the G0–G1 phase [103]. Furthermore, in human inflammation we and others have observed a population of mature CD62L^dim^ neutrophils capable of suppressing T-cell responses through a mechanism which relies on ROS release in an immunological synapse [104]. Recently, similar neutrophils in septic shock patients have been found to express arginase-1 and suppress T-cell functions [105]. Another mechanism by which neutrophils might inhibit T-cell responses is through PD-L1 [106]. Neutrophils isolated from sepsis patients express the surface protein PD-L1, a potent inducer of apoptosis in T cells. The underlying mechanism of PD-L1 expression is an interferon-gamma-dependent process [106]. The PD-1–PD-L1 axis is thought to be an important mechanism in immune suppression in septic patients by inducing lymphocyte apoptosis and monocyte dysfunction [107]. Blocking this axis after the induction of sepsis by administering a PD-1-blocking antibody improved survival in mice [108]. This suppressive mechanism might be protective in tissues with severe inflammatory infiltrates. On the other hand, this process might be unwanted when neutrophils migrate to lymph nodes and engage with adaptive immunity, as has been described under various conditions [109]. In these lymph nodes neutrophils are able to inhibit humoral immune responses through interaction with T and B lymphocytes [109, 110].

## Conclusion

Severe inflammation can result in immune paralysis through various mechanisms. We propose that neutrophils play a central role in this process, either through decreased antimicrobial functions or through direct suppression of (adaptive) immunity. Many experimental studies have been performed addressing the damaging role of neutrophils, which contributes to organ failure in severe inflammation. However, their role in immune paralysis remains understudied. Studies to explore their causative role in susceptibility to infections in animal models of severe inflammation should be designed. Decreased neutrophil antimicrobial functions and their ability to suppress adaptive immunity in vitro should be considered as important patient outcomes. This approach is necessary to increase understanding of the role of neutrophils in immune paralysis leading to detrimental outcome in post-surgical, post-trauma and septic patients.

## Abbreviations

ARDS: acute respiratory distress syndrome
CGD: chronic granulomatous disease
DAMP: damage-associated molecular pattern
IL: interleukin
MAMP: microbe-associated molecular pattern
MDSC: myeloid-derived suppressor cell
NET: neutrophil extracellular trap
ROS: reactive oxygen species
